# Increased [^68^Ga]Ga-SST uptake in the uncinate pancreatic process in new digital PET/CT machine and potential association with clinical and histologic factors in NET patients

**DOI:** 10.1186/s41824-024-00203-x

**Published:** 2024-06-24

**Authors:** Maria Firsova, Giorgio Treglia, Christine Sempoux, Clarisse Dromain, John O. Prior, Niklaus Schaefer, Sarah Boughdad

**Affiliations:** 1 Department of Nuclear Medicine and Molecular Imaging, Lausanne University Hospital, Rue du Bugnon 46, 1011 Lausanne, Switzerland; 2 Faculty of Biology and Medicine, University of Lausanne, Lausanne, Switzerland; 3 Clinic of Nuclear Medicine, Imaging Institute of Southern Switzerland, Ente Ospedaliero Cantonale, Via A. Gallino 12, 6500 Bellinzona, Switzerland; 4 Academic Education, Research and Innovation Area, General Directorate, Ente Ospedaliero Cantonale, Bellinzona, Switzerland; 5 Faculty of Biomedical Sciences, Università della Svizzera Italiana, Lugano, Switzerland; 6 Department of Pathology, Lausanne University Hospital, Lausanne, Switzerland; 7 Department of Radiology, Lausanne University Hospital, Lausanne, Switzerland

**Keywords:** [^68^Ga]Ga-DOTATOC, [^68^Ga]Ga-DOTATATE, [^68^Ga]Ga-SST PET/CT, Uncinate pancreatic process, NET, Physiological uptake, Digital SiPM PET/CT scanner

## Abstract

**Introduction:**

A physiological increase in the uptake of [^68^Ga]Ga-labeled somatostatin analogues ([^68^Ga]Ga-SST) PET tracers has been reported in the uncinate pancreatic process (UP) and might be even higher in latest generation of PET/CT scanners and might be falsely interpreted as NET. We aimed to investigate the uptake of UP in a large population of NET patients who underwent [^68^Ga]Ga-SST PET/CT with digital SiPM detectors. We also explored potential associations between UP uptake and various clinical, imaging, and pathological factors routinely assessed in NET patients.

**Methods:**

We analyzed all consecutive NET patients from July 2018 to June 2022 in this retrospective, single-center study. All patients underwent a [^68^Ga]Ga-SST PET/CT scan on a digital SiPM PET/CT scanner. On visual analysis, we distinguished between normal linear and homogenous UP uptake or abnormal if otherwise. We compared SUV_max/mean_ in patients with normal UP uptake to those with abnormal UP uptake with suspicious NET lesions on contrast-enhanced CT (ce-CT) and according to the site of the primary NET (pancreatic NET vs. other), patient gender (female vs. male) and tumor grade (grade 1–2 vs. 3) using a Mann–Whitney test. We also assessed the correlation between SUV_max/mean_ values in UP with patients’ age, primary NET Ki-67 counting, and its SUV_max/mean_, TLA and MTV values.

**Results:**

We included 131 NET patients with a total of 34 [^68^Ga]Ga-DOTATATE PET/CT and 113 [^68^Ga]Ga-DOTATOC PET/CT scans. An abnormal UP uptake was seen in 32 patients with 65.7% of suspicious NET lesion or extrinsic compression on morphological imaging. Normal UP uptake SUV_max/mean_ were measured in 115 [^68^Ga]Ga-SST scans (78.2%) with normal UP uptake and without suspicious lesion on morphological imaging. We found an average SUV_max_ of 12.3 ± 4.1 for [^68^Ga]Ga-DOTATATE and 19.8 ± 9.8 g/ml for [^68^Ga]Ga-DOTATOC, hence higher than those reported in the literature [SUVmax 5 ± 1.6 to 12.6 ± 2.2 g/ml] with significant difference with abnormal UP uptake and between both PET tracers (both *p* < 0.01). Significant results were a higher UP uptake on [^68^Ga]Ga-DOTATOC in male patients (*p* = 0.02) and significant associations between UP uptake on [^68^Ga]Ga-DOTATOC and SUV_max/mean_ of the primary tumor (ρ [0.337–0.363]; *p* [0.01–0.02]).

**Conclusion:**

We confirmed a higher and very frequent UP uptake in latest SiPM-detector [^68^Ga]Ga-SST PET/CT with an even higher uptake in patients that had [^68^Ga]Ga-DOTATOC PET/CT. SUV_mean/max_ were significantly higher in abnormal UP uptake but there were overlaps with UP SUV values for both [^68^Ga]Ga-SST and a correlation to morphological imaging is crucial. Besides, significant associations between UP uptake and SUV_mean/max_ of the primary NET as well as patients’ gender were seen in the larger cohort of [^68^Ga]Ga-DOTATOC patients suggesting that both physiological and pathological parameters could affect UP uptake.

## Introduction

[^68^Ga]Ga-labeled somatostatin analogues PET tracers are routinely used in patients with neuroendocrine tumors (NET) to detect the primary tumor, to assess the extent of the disease, tumor heterogeneity or before peptide receptor radionuclide therapy to assess patients’ eligibility (Bauckneht et al. 2020). Indeed, it is well-known that NET patients especially those with well-differentiated tumors have a higher expression in somatostatin receptor (SSTR) (Falconi et al. 2012). Various [^68^Ga]Ga-labeled somatostatin analogues ([^68^Ga]Ga-SST) PET tracers are available in routine practice, mainly [^68^Ga]Ga-DOTATATE, [^68^Ga]Ga-DOTATOC and [^68^Ga]Ga-DOTANOC with different affinity for SST receptors (SSTR) (Johnbeck et al. 2014). For instance, [^68^Ga]Ga-DOTATATE present a higher affinity for SSTR-2 whereas [^68^Ga]Ga-DOTATOC has a higher affinity for SSTR-5 (Johnbeck et al. 2014). The increased physiological uptake of [^68^Ga]Ga-SST in the uncinate pancreatic process (UP) has been confirmed in a recent meta-analysis which included six studies with a total of 684 patients and 829 PET/CT scans (Boughdad et al. 2021). This increased [^68^Ga]Ga-SST activity in the head and the UP of the pancreas is related to differences in pathology with in particular a higher density in pancreatic polypeptide cells in comparison to the rest of the gland which express SSTR 1 to 4 (Wittingen and Frey 1974; Brabander et al. 2017). This finding should be accounted for when reporting abnormal finding in NET patients to avoid false positive and unnecessary investigations (Jacobsson et al. 2012). This is especially important since previous studies were done using older generation PET/CT scanners (Al-Ibraheem et al. 2011; Kunikowska et al. 2012) and latest-generation PET/CT with silicon photomultiplier (SiPM), time-of-flight and more efficient reconstruction algorithms that could affect [^68^Ga]Ga-SST uptake of the UP (Ferretti et al. 2018). Therefore, in that setting we investigated the physiological uptake of UP in NET patients that underwent [^68^Ga]Ga-SSTR PET/CT with either [^68^Ga]Ga-DOTATATE or [^68^Ga]Ga-DOTATOC PET tracers on a new digital SiPM PET/CT scan. We also explored associations between UP uptake for both PET tracers and various clinical, imaging, and pathological factors.

## Materials and methods

### Population

In this retrospective and single-center study, we included consecutive NET patients with biopsy proven that had [^68^Ga]Ga-SST PET/CT scan from July 2018 to June 2022. For patients that had multiple [^68^Ga]Ga-SST PET/CT scans during the recruitment period only the first PET/CT for each PET radiotracer was assessed. Patients without a biopsy proven NET or for whom [^68^Ga]Ga-SST PET/CT scan was of poor quality were not included in this study. The study was approved by the local IRB as pointed out below. Patients were recruited with respect to the general consent for the collection of clinical data. PET-CT image acquisition.

### Imaging protocol

All patients had their PET/CT on a SiPM-detector PET/CT (Biograph VISION 600, Siemens Healthineers, Erlangen, Germany) either 60 or 90 min after injection of 2 MBq/kg of [^68^Ga]Ga-DOTATATE or [^68^Ga]Ga-DOTATOC respectively. PET/CT acquisition parameters were as follow: PET acquisition was done at 1.4 mm/s in flow mode with a low-dose CT scan (40mAs, 100 keV, 500 mm FoV, 1.4 mm increment, 2.0 mm slice thickness). PET/CT reconstruction parameters were as follow: TrueX and TOF (ultraHD-PET) reconstruction 4i5S, 440 image size and 2.00 mm Gaussian filter as per our institution protocol, all patients underwent a diagnostic contrast-enhanced CT (ce-CT) at the same time than the PET/CT scan, with arterial and portal acquisition times with technical parameters are as follow: 120 mAs, 120 kV, 1.00 mm slice thickness, Bv38 Kernel, 500 mm FoV, 0.6 mm increment. In patients that had a recent (< 1 month) dynamic contrast-enhanced abdominal MRI (DCE-MRI) or diagnostic ce-CT scan, concomitant ce-CT to [^68^Ga]Ga-SST PET/CT scan was not systematically done as to limit patient radiation exposure.

### PET-CT image analysis

For each patient and for both [^68^Ga]Ga-SST PET tracers, UP uptake was assessed first on visual analysis classifying the uptake as either normal in case of linear and homogenous uptake regardless of its intensity or abnormal if otherwise. All UP uptakes were further investigated on ce-CT (or DCE-MRI) by a trained radiologist (MF), abnormal findings on morphological imaging were collected (lesion suspicious for pancreatic NET, tumor invasion in extensive pancreatic body tumor, extrinsic compression, post-surgical status with missing uptake or local inflammation). For all patients with normal uptake on visual analysis and without abnormal finding on morphological imaging, we measured SUV_max_ and SUV_mean_ (SUV_max/mean_) in the UP. For patients with suspicious findings on morphological imaging such as a lesion suspicious of NET, extensive pancreatic body tumor, or extrinsic compression we also measured SUV_max/mean_ for comparison to patients with normal UP uptake. We also collected data in cases where we were not able to measure UP uptake (post-surgery, etc.).

### Statistical analysis

We used Mann–Whitney (M–W) test to compare SUV_max/mean_ values between patients with normal UP uptake and those with suspicious findings after imaging analysis. We used M-W test to compare UP uptake according to the site of the primary NET (pancreatic NET vs. other), patient gender (female vs. male) and tumor grade (grade 1–2 vs. 3). Additionally, we used M-W test to compare patients’ age between genders for both radiotracers. We also assessed the correlation between SUV_max/mean_ values in UP with patients’ age, primary NET Ki-67 counting and SUV_max/mean_, metabolic tumor volume (MTV) and tumor lesion activity (TLA) measured in the primary NET tumor using Spearman correlation. All statistical analyses were done using IBM SPSS statistics 27.0.1.0 (IBM® SPSS® Statistics) with p-value below 0.05 considered statistically significant.

### Ethics

Our study was done according to the ethical standards stated in the Helsinki declaration and its later amendments. The “Commission cantonale d'éthique de la recherche sur l'être humain” **(**CER-VD) which is our internal review board for ethics approved this retrospective study with informed consent waiver allowing the inclusion of all patients that did not explicitly refuse their consent as per the local legislation at the time of the study **(**registration number: CER-VD-2018-01513).

## Results

**Fig. 1:**
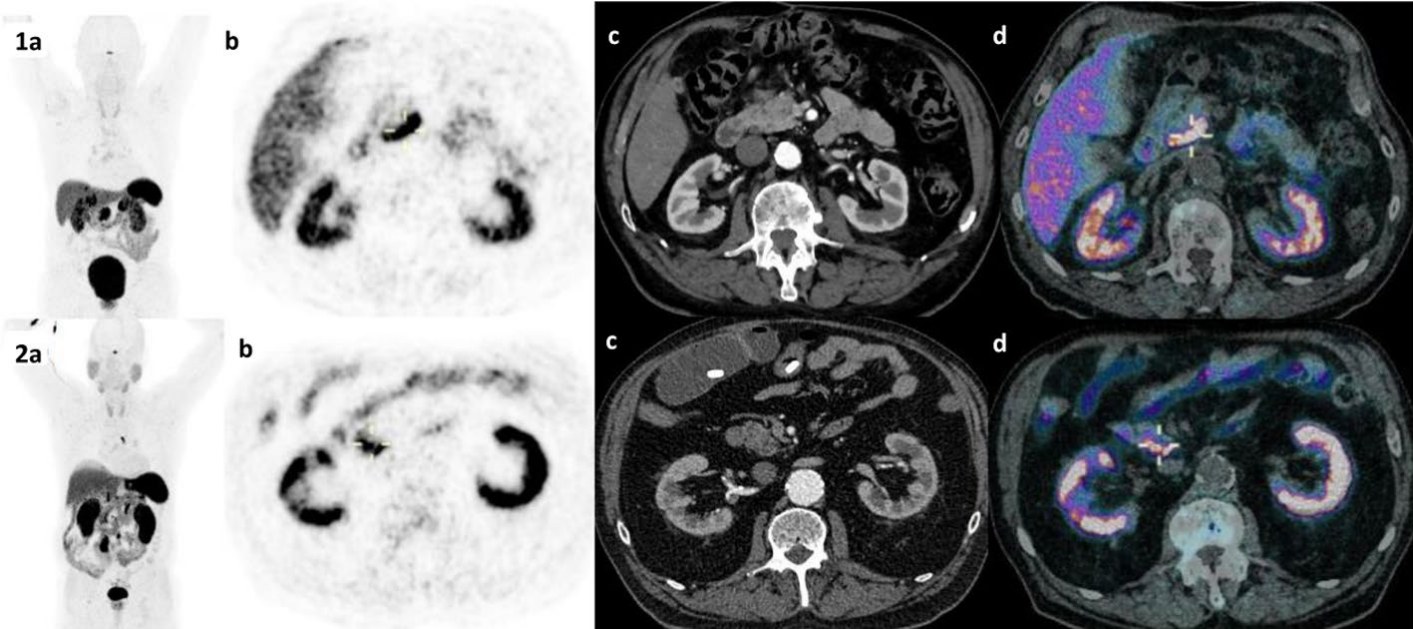
**a** and **b** ^68^Ga-DOTATOC and ^68^Ga-DOTATATE PET/CT images showing normal uptake of the uncinate process of the pancreas in a 84-year man and 79- year man with both grade II mesenteric NET with metastatic spread [top (1) and bottom (2) images respectively]: **a** MIP image, **b** Axial PET slice, **c** Axial CE-CT slice and **d** fused axial PET/CT slice

**Fig. 2:**
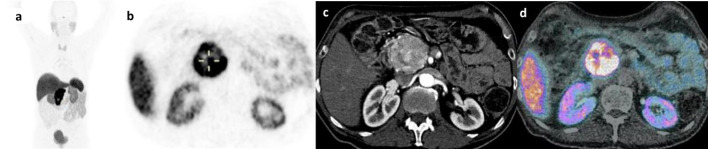
^68^Ga-DOTATATE PET/CT images showing intense uptake of a grade II pancreatic NET of the uncinate pancreatic process in a 73 year man with; **a** MIP image, **b** Axial PET slice, **c** Axial CE-CT slice and **d** fused axial PET/CT slice

**Fig. 3:**
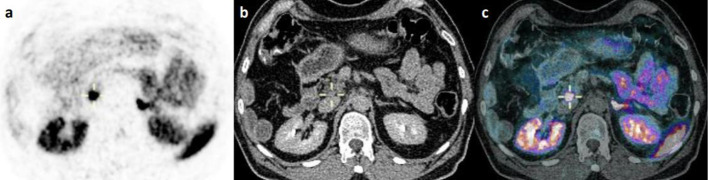
^68^Ga-DOTATATE PET/CT images showing extrinsic compression of the uncinate process of the pancreas by a pre-cave adenopathy in a 55-year man with grade I midgut NET with metastatic spread; **a** Axial PET slice, **b** Axial CT slice and **c** Fused axial PET/CT slice

**Fig. 4:**
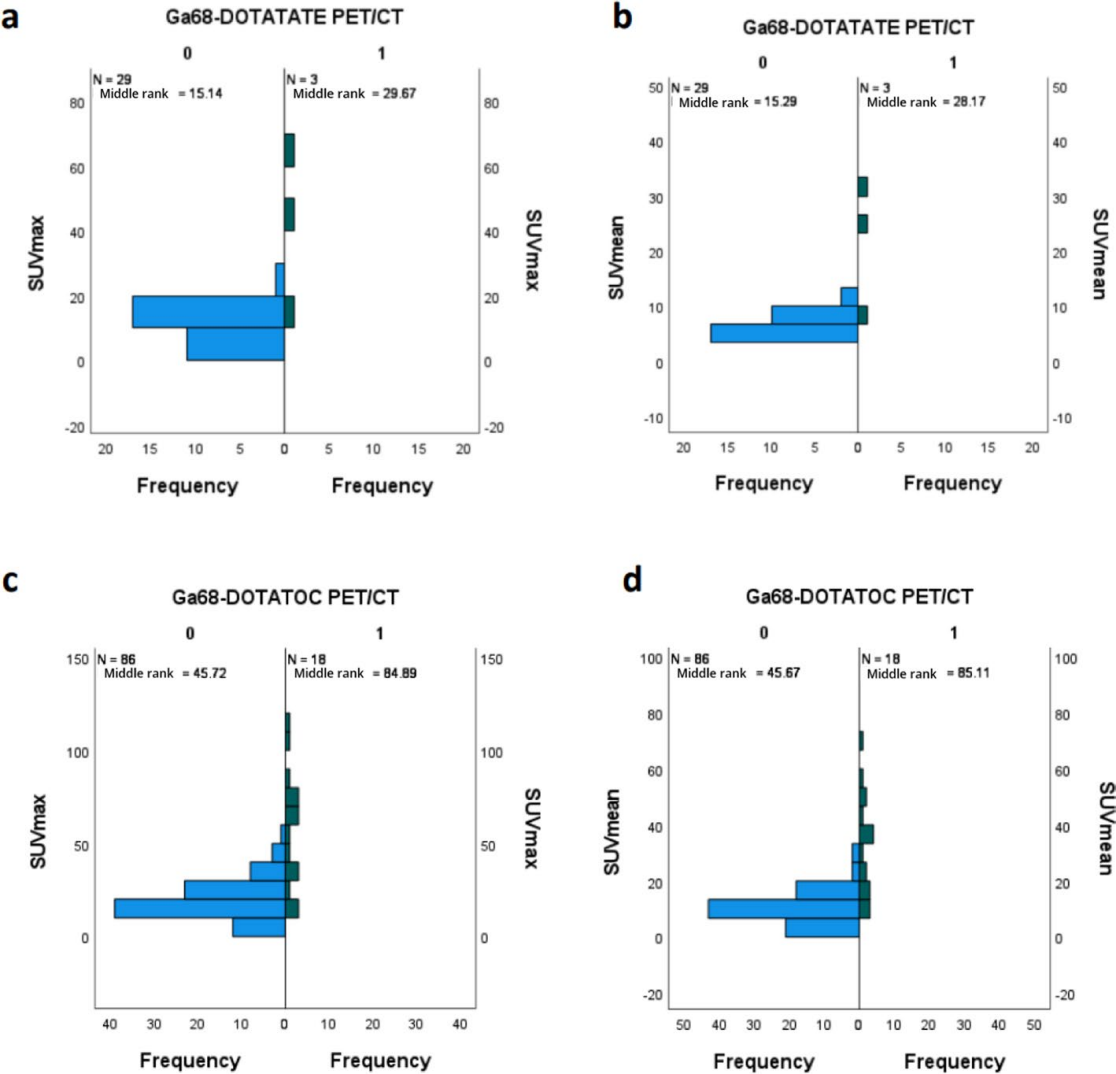
**a**, **b**, **c** and** d** Graphs showing differences in SUVmax (**a**/**c**) and SUVmean (**b**/**d**) values between NET patients that had a ^68^Ga-DOTATATE PET/CT (**a**/**b**) and those who had ^68^Ga-DOTATOC PET/CT (**c**/**d**) scans

**Fig. 5:**
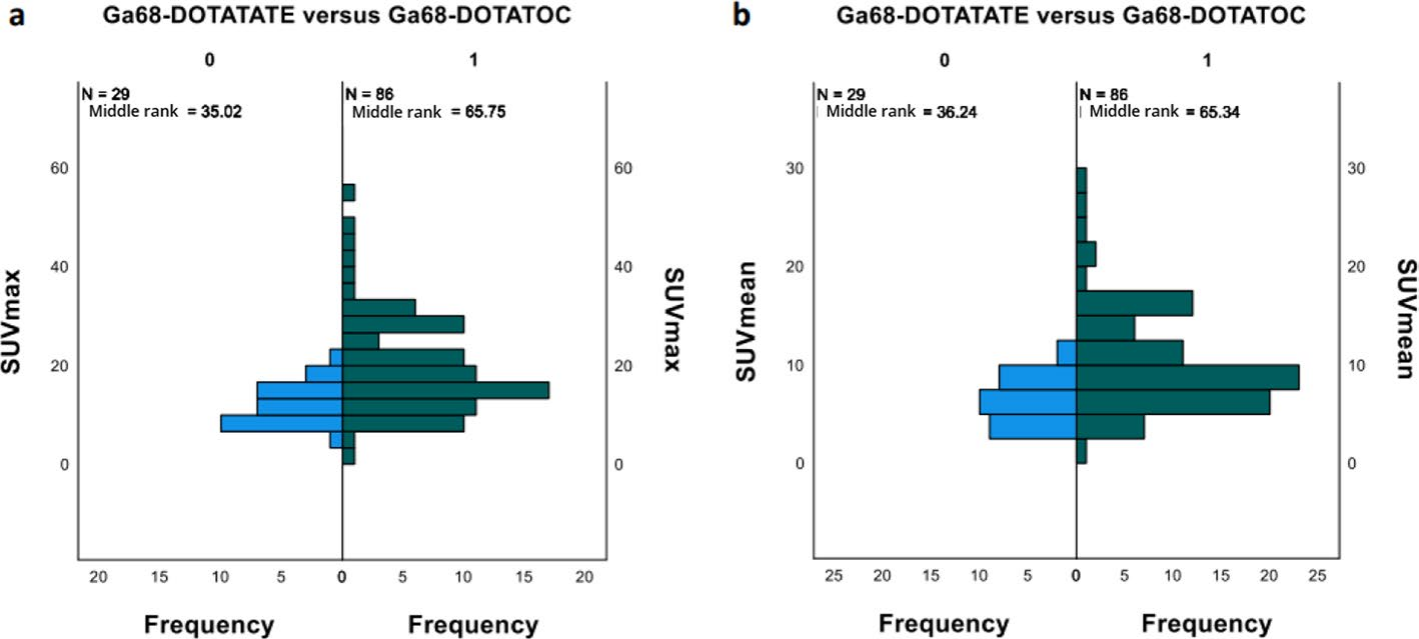
**a** and **b** Graphs showing differences in SUVmax (**a**) and SUVmean (**b**) values between NET patients that had a ^68^Ga-DOTATATE PET/CT and ^68^Ga-DOTATOC PET/CT scans

We included 131 NET patients in this retrospective study with a total of 34 [^68^Ga]Ga-DOTATATE PET/CT and 113 [^68^Ga]Ga-DOTATOC (Fig. 1). Sixteen patients had both a [^68^Ga]Ga-DOTATATE and a [^68^Ga]Ga-DOTATOC PET/CT scans. Patients’ characteristics were similar for both radiotracers with a majority of midgut and pancreatic NETs (Table 1). On the initial visual analysis, we found 5/34 (15%) and 27/113 (23.9%) patients with abnormal UP uptake on a [^68^Ga]Ga-DOTATATE and [^68^Ga]Ga-DOTATOC respectively. On morphological imaging (ce-CT or DCE-MRI) 21 out of those 32 patients (65.7%) corresponded to suspicious NET lesion in or extending to the UP (43.8%) or had extrinsic compression (21.9%; Table 2, Figs. 2, 3). Additionally, 9 out of those 32 (28.1%) patients with abnormal missing uptake had pancreatic surgery. We should also note that one patient had active pancreatitis and one patient had no measurable uptake of the UP on [^68^Ga]Ga-DOTATOC PET/CT. Ultimately, 29 out of 34 patients had a measurable normal uptake of the UP on [^68^Ga]Ga-DOTATATE PET/CT and 86 out of 113 on [^68^Ga]Ga-DOTATOC PET/CT (85.3% and 76.1% respectively; Table 2). All patients with normal UP uptake on visual analysis had no suspicious lesion on morphological imaging (ce-CT or DCE-MRI) When comparing SUV_max/mean_ between patients with normal UP uptake and those with suspicious NET lesion for both [^68^Ga]Ga-DOTATATE and [^68^Ga]Ga-DOTATOC PET/CT scans we found significant differences (Fig. 4a–d; all *p* < 0.001; M-W test). Overall, there were significant differences between [^68^Ga]Ga-DOTATATE and [^68^Ga]Ga-DOTATOC for UP SUV_mean/max_ with significantly higher values in patients that had a [^68^Ga]Ga-DOTATOC PET/CT scan (Table 2, Fig. 5a, b; *p* < 0.001). This was confirmed in the 16 patients had both [^68^Ga]Ga-DOTATATE and [^68^Ga]Ga-DOTATOC PET/CT with 13 patients having normal UP on visual analysis without suspicious lesion on morphological imaging. Indeed, UP uptake was significantly higher when those patients underwent a [^68^Ga]Ga -DOTATOC PET/CT with a mean SUV_max_ of 18.2 ± 7.4 [8.9–32.8] g/ml versus 11.1 ± 2.9 [6.8–16.5] g/ml on a [^68^Ga]Ga-DOTATATE and 9.4 ± 3.9 [4.8–17.2] g/ml versus 6.0 ± 1.5 [3.9–8.5] g/ml for SUV SUV_mean_ (*p* = 0.04 and *p* = 0.007 respectively). There were no significant differences for SUVs according to patient gender for [^68^Ga]Ga-DOTATATE though male patients had higher SUV_mean_ = 7.0 ± 2.2 [3.7–10.2] versus 6.0 ± SD [3.4–9.6] and SUV_max_ = 13.4 ± 4.3 [7.1–20.1] versus 11 ± 3.6 [6.2–16.8] (*p* = 0.7 and *p* = 0.5 respectively). Conversely, significant differences in SUVs were found on [^68^Ga]Ga-DOTATOC PET/CT with higher SUVs in male patients SUV_mean_ = 11.1 ± 4.9 [0.9–24] versus 9.5 ± 5.4 [3.8–28.6] and SUV_max_ = 21.4 ± 9.3 [1.8–45.3] versus 18.1 ± 10.2 [6.4–54] (*p* = 0.03 and *p* = 0.02 respectively). Interestingly, though no significant correlation was between UP SUV_max/mean_ and patients’ age males’ for both radiotracers, we noticed that male patients tended to be older than female patients in the [^68^Ga]Ga-DOTATOC PET/CT cohort with a mean age of 65.4 [30.8–85] years versus 60 [17.4–85.7] years (*p* = 0.17; M-W test). There were no significant differences for UP SUV_max/mean_ according to the location of the primary NET or tumor grade for both radiotracers (*p* > 0.05). Additionally, there were no significant correlations between UP SUV_max/mean_ and Ki67 counting of the primary NET for both radiotracers (*p* > 0.05). Conversely, on [^68^Ga]Ga-DOTATOC PET/CT there were significant associations between UP SUVmax/mean and primary NET SUV_max/mean_ (ρ [0.337–0.363]; *p* [0.01–0.02]) but not with MTV or TLA (*p* > 0.05).

**Table 1:** Patients’ characteristics

Radiotracers	^68^Ga-DOTATATE	^68^Ga-DOTATOC
Number of patients	34	113
Gender	15 Females and 29 males	50 Females and 63 males
Age	63.2 yo [25.1–79.3]	62.2 yo [17.4–85.7]
Tumor grade	Grade 1 = 10 Grade 2 = 14 Grade 3 = 1 Unknown = 9	Grade 1 = 50 Grade 2 = 42 Grade 3 = 5 Unknown = 16
Ki-67%	6.1% [1–35]*	6.7% [1–35]*
Tumor type	Pancreatic NET = 13 Midgut NET = 14 Lung carcinoid tumor = 6 Other = 1	Pancreatic NET = 48 Midgut NET = 39 Lung carcinoid tumor = 12 Other = 14
TNM status	Metastatic spread = 24 LN involvement = 3 Isolated tumor = 6 Unknown = 1	Metastatic spread = 60 LN involvement = 14 Isolated tumor = 37 Unknown = 2

*[minimum–maximum value]

**Table 2:** Comparison of imaging findings on visual analysis and SUVmean/SUVmax measurements between ^68^Ga-DOTATATE and ^68^Ga-DOTATOC PET tracers

^68^Ga-SST PET tracers		^68^Ga-DOTATATE Mean ± SD [min–max]	^68^Ga-DOTATOC Mean ± SD [min–max]
Normal uptake on visual analysis		29	86
Abnormal uptake or missing		5 (2 absent post-surgery/1 LN extrinsic compression/2 Tumor extension*)	27 (7 absent post-surgery/6 extrinsic compression/12 Tumor with 1 tumor extension*/1 non measurable and 1 pancreatitis)
Uncinate process of the pancreas uptake	SUVmax	12.3 ± 4.1 [6.2–20.5]	19.9 ± 9.8 [1.8–54]
	SUVmean	6.6 ± 2.1 [3.4–10.2]	10.4 ± 5.2 [0.9–28.6]
Primary NET uptake	SUVmax	37.7 ± 25.4 [0–77]	51.9 ± 42.9 [3.4–290]
	SUVmean	21.1 ± 14.6 [0–43]	29.8 ± 25.2 [1.9–167.7]

*Patients with large pancreatic body NET that infiltrated the UP

## Discussion

In this study we analyzed the uptake of the uncinate pancreatic process in a large population of NET patients using [^68^Ga]Ga-DOTATATE or [^68^Ga]Ga-DOTATOC on a digital SiPM PET/CT scanner. To our knowledge this is the first analysis being done in modern state-of-the-art ultrafast PET/CT scanners. We found that UP uptake was measurable in most patients that had [^68^Ga]Ga-SST PET/CT scan, with 85.3% normal and measurable UP uptake on [^68^Ga]Ga-DOTATATE and 76.1% on [^68^Ga]Ga-DOTATOC. In our study, UP uptake was first assessed on visual analysis for both radiotracers and ultimately classified as either normal or abnormal based on ce-CT results as described above, confirming the usefulness of combining morphological and metabolic information (Bauckneht et al. 2020). Indeed, most patients with abnormal UP uptake on visual analysis that is 27.1% had a lesion suspicious of NET on ce-CT or presented with an extrinsic compression. As expected, SUV_max/mean_ values in abnormal UP, hence suspicious NET lesions were significantly higher than in normal UP but overlaps between SUV_max/mean_ values were seen with a wide variation in SUVs values in UP, ranging for instance from 1.8 to 54 for SUV_max_ in patients that underwent a [^68^Ga]Ga-DOTATOC PET/CT. In that setting, a combined reporting of findings on CT, especially ce-CT remained essential to confirm the absence of suspicious lesion especially additional NET lesion in agreement with recent guidelines (Bauckneht et al. 2020). Looking at the comparison of UP uptake according to the PET tracers we found significantly higher SUV_max/mean_ values in patients that had [^68^Ga]Ga-DOTATOC PET/CT scans. This finding could be explained by the differences in affinity for SST receptors previously reported in the literature but also in differences between acquisition times (Calabrò et al. 2020; Poeppel et al. 2011; Kabasakal et al. 2012). Indeed, per our institution protocols there was a difference in acquisition time after radiotracer injection between [^68^Ga]Ga-DOTATATE and [^68^Ga]Ga-DOTATOC with an earlier acquisition at 60 min for the former and 90 min for the latter, which likely affected the intensity of the uptake, hence SUV_max/mean_ values for both UP and the primary NET lesion (Novruzov et al. 2021; Nakamoto et al. 2016). In the subgroup of 16 patients that had both a [^68^Ga]Ga-DOTATATE PET/CT and a [^68^Ga]Ga-DOTATOC significantly higher SUV_max/mean_ values were also found in the latter. Looking at the immunochemistry, it has been reported that SSTR2 expression is low in pancreatic peptide cells present in the UP which might explain the lower uptake of [^68^Ga]Ga-DOTATATE which has a higher affinity for SSTR2 in comparison to [^68^Ga]Ga-DOTATOC (Portela-Gomes et al. 2000). However, SSTR5 which present a higher affinity for [^68^Ga]Ga-DOTATOC was almost absent in pancreatic polypeptide cells and thus could not explain the differences in uptake (Portela-Gomes et al. 2000). We should however mention that those immunochemistry findings were reported in an older study using rabbit polyclonal antibodies and though they were specific for each human STTR, it might be interesting to reassess those findings with current methods (Portela-Gomes et al. 2000). Nevertheless, those differences should be accounted for in NET patients during the follow-up as they tend to have multiple [^68^Ga]Ga-SST PET/CT scans and the PET tracer might vary within and across institutions. Besides, we investigated which factors could influence UP uptake in NET patients as this is the main population of patients referred for [^68^Ga]Ga-SST PET/CT scans in routine practice (Bauckneht et al. 2020; Chen et al. 2018). We did not find significant correlation between UP uptake and patients’ age in both [^68^Ga]Ga-SST tracers though due to an atrophy of the acinar tissue with age leading to a higher density of pancreatic polypeptide cells’ islets with an appearance of pseudo-hyperplasia in the UP which could hypothetically expect an increase [^68^Ga]Ga-SST UP uptake (Portela-Gomes et al. 2000). By contrast, patients’ gender did significantly affect UP uptake with significantly higher SUV_max/mean_ UP values in male patients in comparison to female patients on [^68^Ga]Ga-DOTATOC PET/CT scans. It would be interesting to assess if it relates to difference of SST receptor on immunohistochemistry according to patients’ gender though no data was available in the literature. Additionally, we should also point out that although it was not significant male patients also tended to be older than female patients and maybe in a larger cohort of patients a significant association between UP uptake could be seen (Wittingen and Frey 1974). The lack of difference found on [^68^Ga]Ga-DOTATATE might be also explained by the small number of patients in this cohort. Besides, the only significant associations found with SUV_max/mean_ in the UP were moderate but significant correlations with SUV_max/mean_ in the primitive NET. The significance of this association is still unclear, but it would be interesting to confirm this association in future cohorts of NET patients.

We previously investigated the uptake of UP in a recent meta-analysis which assessed the incidental uptake of UP and included 4 studies with [^68^Ga]Ga-DOTATOC and [^68^Ga]Ga-DOTATATE tracers with 59.1% and 13.9% incidental UP uptake respectively (Boughdad et al. 2021; Jacobsson et al. 2012; Al-Ibraheem et al. 2011; Kunikowska et al. 2012; Mapelli et al. 2014). The lower incidence of UP uptake in those studies might be explained by the difference in PET/CT scanner technology as all those studies were done in older generation whereas all scans in our study were acquired in a new digital SiPM PET/CT (Boughdad et al. 2021). Indeed, a detectable uptake of UP was visible and measurable on all patients with both [^68^Ga]Ga-SST PET tracers except for 11 patients including 9 patients that surgery with a total 78.2% of physiological uptake and 14.3% abnormal uptake with a suspicious NET lesion on ce-CT. Looking, at SUV values we had in the [^68^Ga]Ga-DOTATOC cohort a mean SUV_max_ value of 19.9 ± 9.8 g/ml versus SUV_max_ = 9.8 ± 12.5 g/ml in the study by Al-Ibraheem et al. (Boughdad et al. 2021; Al-Ibraheem et al. 2011). Similarly, in the [^68^Ga]Ga-DOTATATE cohort the mean SUV_max_ value was of 12.3 ± 4.1 g/ml versus in the study SUV_max_ 9.2 ± 3.3 g/ml in the study by Kunikowska et al. (Boughdad et al. 2021; Kunikowska et al. 2012). The increased detection of UP uptake on [^68^Ga]Ga-SST PET/CT scans is likely explained in our study by the implementation of better detector resolution, faster time-of-flight, and better sensitivity of the latest-generation of SiPM-detector PET/CT leading to improved recovery coefficient and increased uptake seen both on visual analysis and semi-quantitative SUVs measurements (Ferretti et al. 2018; Calabrò et al. 2020; Ashrafinia et al. 2017). Thus, as previously mentioned, the intensity of the UP uptake should not be used alone to distinguish between physiological and pathological UP uptake but in addition to ce-CT. Nevertheless, our findings should be useful to nuclear physicians in routine practice by providing a range for SUV_mean/max_ values in normal UP for [^68^Ga]Ga-DOTATATE and [^68^Ga]Ga-DOTATOC PET/CT in the absence of morphological abnormalities on ce-CT, as SiPM-detector PET/CT scanners are spreading across institutions. UP uptake may no longer have to be considered as an incidental finding but a caveat that should be accounted for when reporting [^68^Ga]Ga-SST PET tracers scans in NET patients as it was seen in 78.2% of the patients in our study. The combined assessment of molecular imaging and ce-CT is essential to guide additional investigations in case of visually abnormal or excessively high [^68^Ga]Ga-SST UP uptake especially if a biopsy is required.

We should also mention some limitations in our study. The first limitation was the smaller numbers of patients who had [^68^Ga]Ga-DOTATATE in comparison to a [^68^Ga]Ga-DOTATOC PET/CT, but patients’ characteristics were similar with predominantly male patients with pancreatic and midgut NETs, with similar Ki-67 counting and comparison of UP SUV_max/mean_ was statistically significant. Indeed, only patients that had their scans on a new SiPM-detector PET/CT were included and we had a change in [^68^Ga]Ga-SST PET tracer used in routine practice in our institution, leading to a limited time of 9 months for the recruitment of patients that had [^68^Ga]Ga-DOTATATE PET tracer before we started using [^68^Ga]Ga-DOTATOC. Indeed, [^68^Ga]Ga-DOTATOC was produce by our radiopharmacists in a dedicated facility in our institution. Secondly, in patients with abnormal UP uptake and with suspicious NET lesions seen on CE-CT no follow-up pathological data was collected as most patients had a history of biopsy proven NET and were at a metastatic stage at the time of PET/CT, that was 70.6% of the patients that had a [^68^Ga]Ga-DOTATATE PET/CT scan and 53.1% of the patients that had [^68^Ga]Ga-DOTATOC PET/CT scan. Finally, several parameters were thought to be outside of the scope of this study and were not explored such as the impact on the Krenning score according to the tumor grade, presence of a tumor predisposition syndrome and whether the primary NET lesion was secretory or not. Indeed, the data collection was retrospective and some of this information were missing but it might be interesting to investigate their influences on prospective studies.

## Conclusion

We confirmed the higher and very frequent UP uptake in new SiPM-detector PET/CT scanners for both [^68^Ga]Ga-DOTATATE and [^68^Ga]Ga-DOTATOC in comparison to older generation PET/CT scanners. This is of interest, as we found that SUV values could be very high without any suspicious lesions seen on morphological imaging. Indeed, though abnormal UP uptake with suspicious NET lesions on ce-CT had significantly higher SUV_max/mean_ than those measured in the UP there were overlaps for both [^68^Ga]Ga-SST tracers. Thus, a high SUV_max_ value in the UP is not sufficient to suspect the presence of NET and a correlation to morphological imaging is crucial. This should be clearly understood by nuclear medicine physician while reporting [^68^Ga]Ga-SST PET/CT imaging to limit unnecessary investigations in NET patients. Besides, significant associations between UP uptake and SUV_max/mean_ of the primary NET as well as patients’ gender were found in the largest cohort of [^68^Ga]Ga-DOTATOC patients suggesting that both physiological and pathological parameters could affect UP uptake.

## Data Availability

The datasets used and/or analyzed during the current study are available from the corresponding author on reasonable request.

## Abbreviations

[^68^Ga]Ga-SST: [^68^Ga]Ga-labeled somatostatin analogues
ce-CT: Contrast-enhanced CT
DCE-MRI: Dynamic contrast-enhanced abdominal–Magnetic resonance imaging
MTV: Metabolic tumor volume
NET: Neuroendocrine tumors
SSTR: Somatostatine receptor
SiPM: Silicon photomultiplier
TLA: Tumor lesion activity
UP: Uncinate pancreatic process
